# Zebrafish Bioassay for Screening Therapeutic Candidates Based on Melanotrophic Activity

**DOI:** 10.3390/ijms22179313

**Published:** 2021-08-27

**Authors:** Ted I. Hong, Kyu-Seok Hwang, Tae-Ik Choi, Gunnar Kleinau, Patrick Scheerer, Jeong Kyu Bang, Seung-Hyun Jung, Cheol-Hee Kim

**Affiliations:** 1 Department of Biology, Chungnam National University, Daejeon 34134, Korea; tihong10@gmail.com (T.I.H.); c860523@naver.com (T.-I.C.); 2 Drug Discovery Platform Research Center, Korea Research Institute of Chemical Technology, Daejeon 34114, Korea; kshwang@krict.re.kr; 3 Group Protein X-ray Crystallography and Signal Transduction, Institute of Medical Physics and Biophysics, Charité-Universitätsmedizin Berlin, Corporate Member of Freie Universität Berlin and Humboldt-Universität zu Berlin, D-10117 Berlin, Germany; gunnar.kleinau@charite.de (G.K.); patrick.scheerer@charite.de (P.S.); 4 Division of Magnetic Resonance, Korea Basic Science Institute, Ochang, Cheongju 28119, Korea; bangjk@kbsi.re.kr; 5 Department of Applied Marine Bioresource Science, National Marine Biodiversity Institute of Korea, Seocheon 33662, Korea

**Keywords:** zebrafish, bioassay, melanophore, autism spectrum disorder, alpha-melanocyte stimulating hormone, melanotan-II

## Abstract

In this study, we used the zebrafish animal model to establish a bioassay by which physiological efficacy differential of alpha-melanocyte-stimulating hormone (α-MSH) analogues could be measured by melanosome dispersion in zebrafish larvae. Brain-skin connection research has purported the interconnectedness between the nervous system and skin physiology. Accordingly, the neuropeptide α-MSH is a key regulator in several physiological processes, such as skin pigmentation in fish. In mammals, α-MSH has been found to regulate motivated behavior, appetite, and emotion, including stimulation of satiety and anxiety. Several clinical and animal model studies of autism spectrum disorder (ASD) have already demonstrated the effectiveness of α-MSH in restoring the social deficits of autism. Therefore, we sought to analyze the effect of synthetic and naturally-occurring α-MSH variants amongst different species. Our results showed that unique α-MSH derivatives from several fish species produced differential effects on the degree of melanophore dispersion. Using α-MSH human form as a standard, we could identify derivatives that induced greater physiological effects; particularly, the synthetic analogue melanotan-II (MT-II) exhibited a higher capacity for melanophore dispersion than human α-MSH. This was consistent with previous findings in an ASD mouse model demonstrating the effectiveness of MT-II in improving ASD behavioral symptoms. Thus, the melanophore assay may serve as a useful screening tool for therapeutic candidates for novel drug discovery.

## 1. Introduction

Melanocortins are a family of peptides initially discovered as factors involved in the regulation of skin color in frogs. Etymologically, melanocortins encompass the subgroups, melanotropin and corticotropin, based on their mutual tetrapeptide core sequence [1]. Melanocortin peptides derive from the protein precursor, pro-opiomelanocortin (POMC) [2]. POMC expression has been detected in various central and peripheral locales, such as the hypothalamus, skin, and placenta [3]; however, the major site of expression is the pituitary gland [4]. Tissue-specific post-translational processing of POMC preproprotein produces several mature hormones including four distinct melanocortin peptides—adrenocorticotrophic hormone (ACTH), α-melanocyte-stimulating hormone (MSH), β-MSH, and γ-MSH. Among these, α-MSH acts as the endogenous ligand for melanocortin receptor 1 (MC1R), which is expressed on the cell membrane of teleost melanophores and plays a key role in skin pigmentation [5,6]. Additionally, research has demonstrated that α-MSH analogues show effectiveness as a pharmacological therapeutic for ASD [7,8].

ASD refers to a group of neurodevelopmental disorders which encompasses a wide continuum of severities and symptoms. The disorder was first systematically described in a landmark paper accounting clinical manifestations of eleven child subjects, who exhibited a common propensity for social isolation [9]. ASD symptoms are classified into two core domains—social communication impairment and repetitive behavior [10]. Social deficits are the defining feature of ASD; however, they are often cited as the most challenging aspect of the disorder by parents and caretakers [11]. Nevertheless, none of the currently approved ASD medications are effective in improving ASD’s core social symptoms, and none have yet been approved that specifically treat the behavioral symptoms of ASD [12,13]. However, the steady rise of ASD prevalence has stimulated research efforts, which have yielded important insights regarding epidemiology of the disorder and have offered hope in developing future ASD therapeutics to counteract the social burden on families and caretakers.

In a double-blind, placebo-controlled trial of 14 autistic children, a synthetic form of ACTH (ACTH 4-9), comprised entirely of an upstream region containing α-MSH, decreased stereotypic behaviors while significantly improving social interaction between child and experimenter [8]. The same compound showed effectiveness in adults by increasing interpersonal awareness among those with intellectual disability [14]. Research involving ASD animal models have also demonstrated the effectiveness of α-MSH-based derivatives in improving social behavior; for example, one study found that α-MSH analogues could promote social bonding in a sample of prairie voles [15]. Therefore, the development of tools to screen α-MSH-derived compounds would contribute to the development of novel therapeutics to effectively manage ASD behavioral symptoms.

To this end, we developed the zebrafish bioassay, which measures the pharmacological efficacy of α-MSH derivatives based on the extent of melanophore dispersion. Zebrafish possess several features suitable towards this end: (1) they are social animals amenable to behavioral experiments that show complex behaviors ranging from learning and cognition to aggression and anxiety [16]; (2) they represent more than 80% of human disease genes in their genome with close similarity to the brain structures of humans [17,18]; and (3) they have been successfully employed to validate candidate genes involved in head formation/neurogenesis, intellectual disability, microcephaly, and ASD [19,20,21]. In addition, zebrafish melanophore pigment cells exhibit dynamic responses to certain sets of chemical compounds. Therefore, we compared the physiological effect of α-MSH sequence variants measured by the extent of melanophore dispersion upon application of α-MSH analogues. Our findings showed that the synthetic α-MSH analogue, melanotan-II (MT-II), exhibited the highest potency among the derivatives tested, which corresponded to high efficacy in behavioral studies conducted in a mouse model of ASD [7].

## 2. Results

**Figure 1:**
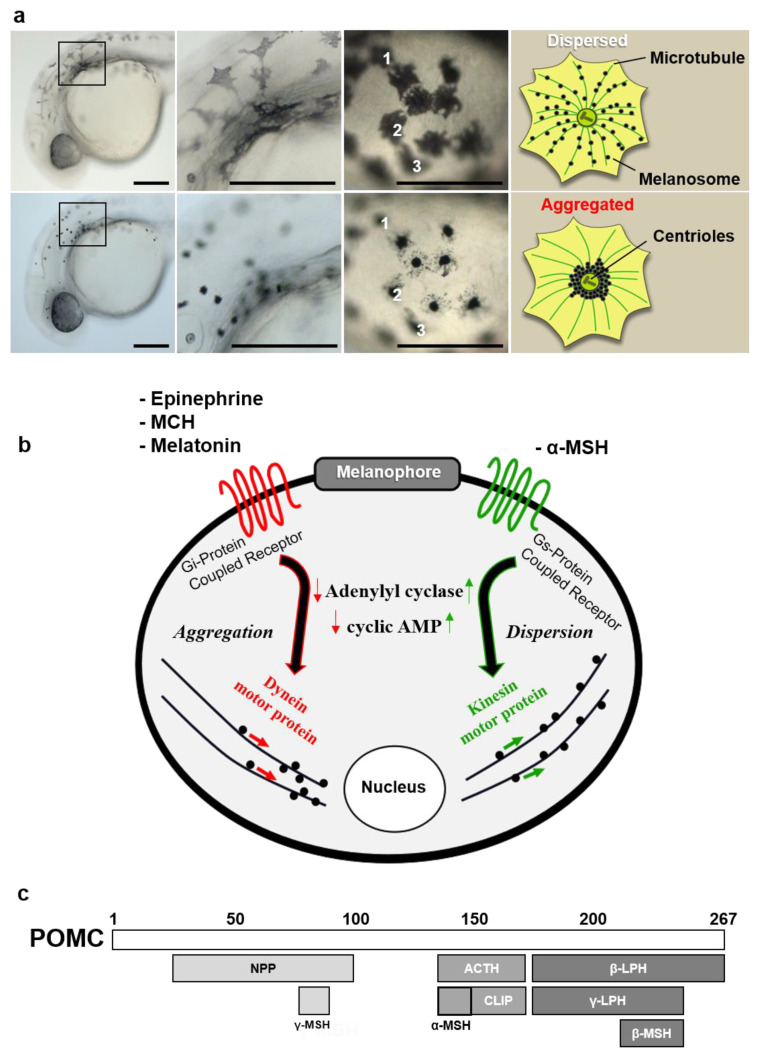
Characteristic features of zebrafish melanophore physiology. (**a**) High-resolution magnification of larval melanophores before and after treatment with the aggregating agent. Numbers (1, 2, 3) indicate individual melanophores two minutes after melatonin treatment. Schematic describes movement of melanosomes during aggregation process. Scale bars: 100 µm. (**b**) Diagram of melanophore pigment cell showing major intracellular events following receptor-ligand binding of aggregating/dispersive agents. (**c**) Schematic showing POMC gene product and derivative molecules generated by post-translational processing. Abbreviations: mch: melanin-concentrating hormone, npp: *N*-terminal peptide of proopiomelanocortin, clip: corticotropin-like intermediate peptide, lph: lipotropin.

Utilizing aggregative and dispersive features of zebrafish larval melanophores, we devised a biological assay to measure physiological potency by measuring the extent of melanophore dispersion from an initially aggregated state. Larval melanophores normally rest in a state of melanosome dispersion. In response to chemicals such as melatonin, these dark, melanin-storing organelles travel along microtubules from the periphery toward the microtubule organizing center located near the nucleus (Figure 1a). Distinct sets of compounds act via specific G-protein-coupled receptors (GPCRs), setting off signaling cascades that alter intracellular concentration of cyclic adenosine monophosphate (cAMP) and activate the motor proteins involved in melanosome transport (Figure 1b). One of these compounds, α-MSH, is a 13-amino acid proteolytic cleavage product of ACTH known to induce melanosome dispersion via G_s_-protein-coupled MC1R and kinesin motor proteins (Figure 1c); on the other hand, compounds such as melatonin signal through the G_s_-protein-coupled receptor downregulating adenylyl cyclase and decrease intracellular cAMP levels, leading to an aggregation response.

**Figure 2:**
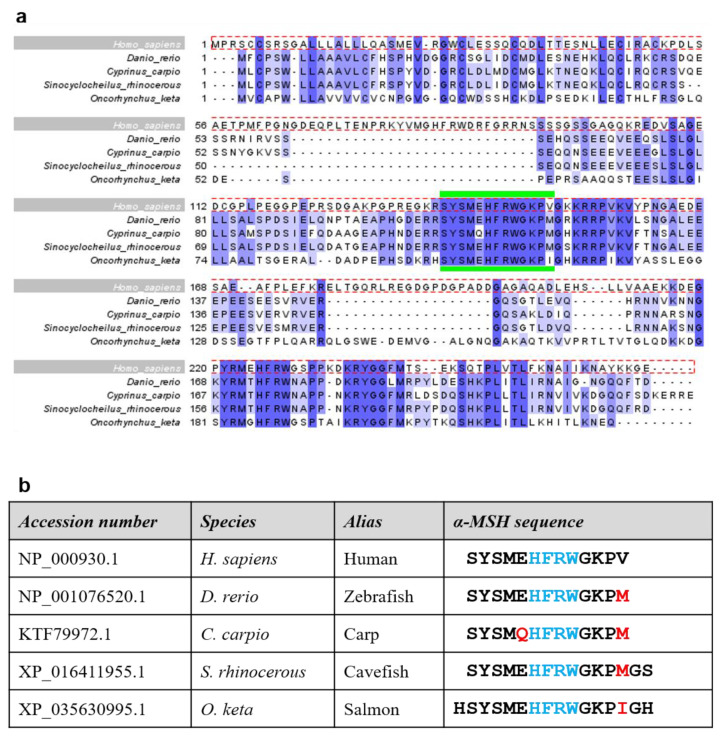
Sequence analysis and alignment of naturally-occurring α-MSH variants. (**a**) Clustal Omega alignment of four α-MSH variants in different species with human sequence as reference (dotted red box). Percentage Identity color scheme. The region demarcated by green bars indicates the 13-amino-acid α-MSH peptide. (**b**) Table listing the accession number of each α-MSH form used in the melanophore assay, including aliases for each species. The α-MSH sequences indicate the sequence of generated peptides; red letters indicate amino acid variations within the α-MSH peptide, while blue represents the HFRW motif.

Given the relevance of α-MSH in ASD therapy, we devised a protocol to assess the potency of derivative compounds. Our aim was to develop an assay method that was sensitive to subtle changes in the amino acid sequence of test peptides. Thus, we started our analysis by searching for naturally-occurring sequence variants of α-MSH among different species with unique ecological/physiological features; for example, eye- and pigment-less *Sinocyclocheilus rhinocerous* cavefish or anadromous (i.e., able to live in both fresh and salt water environments) and body-color-changing chum salmon (*Oncorhynchus keta*). Our analysis revealed that α-MSH sequences were identical among mammals, while substantial variation existed among fish species. Ultimately, three fish species were selected in addition to human and zebrafish, and multiple sequence alignment of the POMC precursor protein and α-MSH was conducted (Figure 2a). From the sequence information, α-MSH variant sequences were custom synthesized for analysis using the melanophore assay (Figure 2b).

**Figure 3:**
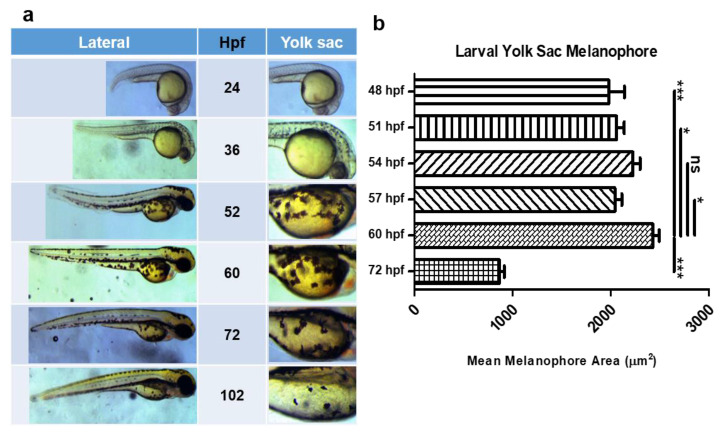
Yolk sac melanophores of developing zebrafish larvae. (**a**) Table of images depicting zebrafish larva developmental stages according to the number of hours post fertilization (hpf) with magnification of the yolk-sac region. (**b**) Quantification of average area of yolk sac melanophores at 3 h intervals starting from 48 hpf to 60 hpf for wild-type zebrafish. One-way ANOVA with post-hoc Dunn’s multiple comparison test was used for statistical significance between groups; data represented as mean ± SEM; * *p* < 0.05, *** *p* < 0.001, ns = not significant.

**Figure 4:**
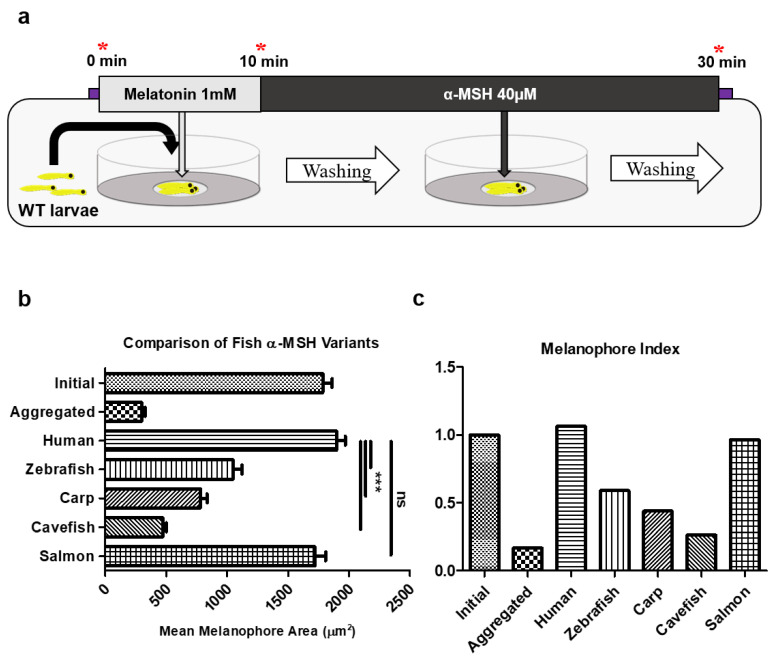
Melanophore dish assay comparing α-MSH variants from different species. (**a**) Schematic of melanophore dish assay with wild-type (WT) larvae. Red asterisks indicate imaging timepoints. (**b**) Results of melanophore dish assay comparing mean melanophore area (*n* = 30–50 melanophores from 3–5 larvae) calculated after induction of melanosome dispersion by treatment of the respective α-MSH peptide. One-way ANOVA with post-hoc Dunn’s multiple comparison test was used for statistical significance between groups; data represented as mean ± SEM; *** *p* < 0.001, ns = not significant. (**c**) Normalization of mean melanophore area by initial area of the control group (see Equation (1)).

Throughout the course of the study, the melanophore assay underwent two phases of development with the introduction of equipment to streamline the protocol. The first iteration is referred to as the “dish protocol” or “dish assay” (Figure 4a). At the start of the experiment, we first determined the yolk-sac region of zebrafish larvae as the field of observation for melanophore measurement (Figure 3a). Although melanophore development occurs throughout the body at early stages of development, melanophores in the yolk-sac region are noticeably abundant. In addition, this region provides a convenient anatomical demarcation to identify the melanophore pool of the experiment. Observation of yolk sac melanophore development showed steady growth of melanophore size and quantity until about 2–3 days post fertilization (dpf), after which point melanophores eventually disappeared from the yolk sac almost entirely by 4dpf (Figure 3a,b). Thus, 48 h post fertilization (hpf) to 60 hpf zebrafish larvae were selected for the assay experiment.

(1) $$Melanocyte\;Index\;\left(MI\right)=Mean\;area\;of\;\frac{Experimental\;group}{Untreated\;control\;group}.$$

Using the human form as reference standard, we compared the physiological effect of the four fish α-MSH variants using the melanophore dish assay. From an initially aggregated state, each of the four unique fish peptides produced different degrees of melanosome dispersion (zebrafish: 1051.04 μm^2^, carp: 781.03 μm^2^, cavefish: 472.68 μm^2^, and salmon: 1721.08 μm^2^), although none exceeded dispersion of the human form (1898.20 μm^2^) (Figure 4b). To visualize the extent of melanosome dispersion relative to the original average size of melanophores at the start of the experiment, the melanocyte index was formulated (Equation (1)) and calculated (Figure 4c), showing differential dispersion of test derivatives.

**Figure 5:**
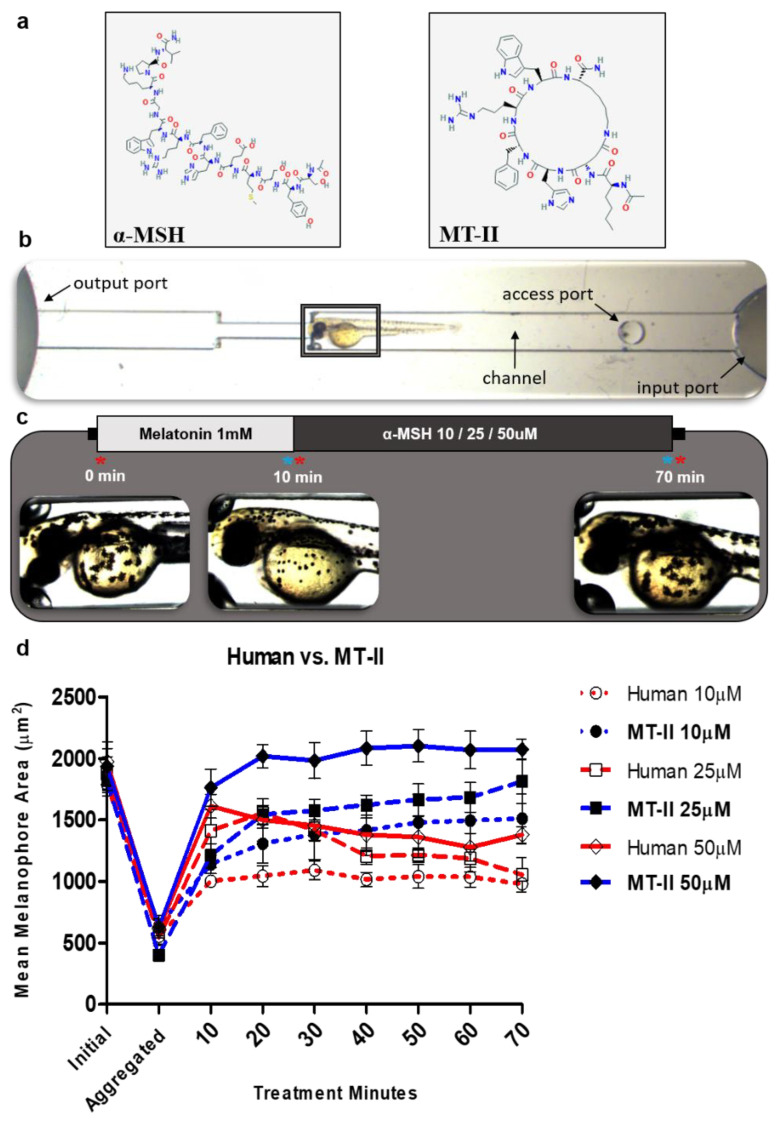
Melanophore restriction array assay comparing human α-MSH and MT-II. (**a**) Diagram representing the two-dimensional structure of α-MSH and MT-II. (**b**) Image of ZEBRA (zebrafish restriction array) device with major structural features labeled. The gray box indicates the region of magnification at imaging steps. (**c**) Protocol of melanophore restriction array assay. Red asterisks indicate imaging timepoints, while blue asterisks indicate timepoints for washing. (**d**) Curve shows the change of mean melanophore area over the treatment time for each peptide and concentration. Each curve aggregates the average of three trials (*n* = 40–50 melanophores from 4–5 larvae for each trial). Data represents mean ± SEM.

In order to further demonstrate the functionality of the assay, we conducted literature search for prospective target compounds demonstrating relevance as an ASD therapeutic. Results yielded a synthetic α-MSH analogue known as melanotan-II (MT-II) that had already been shown to alleviate social behavioral deficits in an ASD mouse model. Intraventricular injections of MT-II to maternal-immune activation (MIA) mouse model of ASD rescued social behavior metrics in the study [7]. The seventh residue, circular peptide, differed noticeably from the original α-MSH molecule in both length and structural configuration (Figure 5a). The synthetic analogue has demonstrated the ability to cross the blood-brain barrier (BBB), as can α-MSH; however, MT-II has a reported half-life of 1–2 h, which is more than triple the half-life of α-MSH (20–25 min) [22]. By introducing a microfluidic restriction array device [23] and eliminating labor-intensive mounting steps (restriction array protocol) (Figure 5b,c), imaging capability was greatly expanded for the duration of the assay, thereby, allowing observation of the effects of compound stability on melanophore dispersion. Comparing melanosome dispersion of MT-II to human α-MSH by the restriction array protocol, we found that average melanophore area tended to decrease over time for the two highest concentrations of human α-MSH tested (25 and 50 μM); on the other hand, the average area for MT-II either maintained at high levels or showed an increasing effect over the course of the experiment (Figure 5d). Using the restriction array protocol, MT-II was found to effect greater melanosome dispersion than human α-MSH, demonstrating the sensitivity of the melanophore assay to detect and differentiate low potency compounds as well as to accurately measure compounds of greater pharmacological efficacy, such as MT-II.

**Figure 6:**
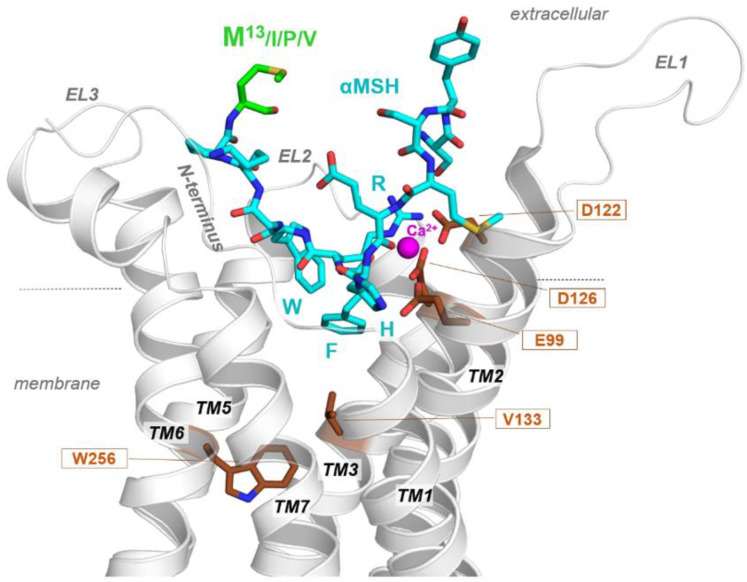
Molecular model of α-MSH and zebrafish MC1R complex. The MC1R (carton representation) binds α-MSH in a cavity between the extracellular loops (ELs) and the transmembrane helices (TMs). Visualized are amino acids (sticks) covering the ligand binding site and are highly important for ligand and co-factor calcium (Ca^2+^) binding. All α-MSH ligand variants from different species share a conserved core motif (HRFW) involved in receptor-ligand contacts. The α-MSH C-terminus with position 13 (M13) is not in direct contact with the receptor as supposed by MC4R/α-MSH or MC1R/α-MSH complexes.

In order to investigate whether α-MSH variants from different species have potentially diverse impact on receptor binding, we designed a structural model of the zebrafish MC1R in complex with a variant form of zebrafish α-MSH. For this purpose, we used the recently determined MC4R/NDP-αMSH complex as a structural template [24]. This MC1R complex-model (Figure 6) revealed that the amino acid at position 13 of the ligand is likely not directly involved in contact with the MC1R (nor between human α-MSH and human MC4R). This is in agreement with recent insights from a human MC1R/α-MSH complex [25]. In addition, we assume that any variation in this position does not alter the ligand structure itself, as in high-affinity MSH subtypes (e.g., β-MSH) this position is also variable [26] as observed here in α-MSH variants of different species (Figure 2b). These findings and predictions are consistent with the results of our in vivo assay, which demonstrates the biological activity of the tested ligands, albeit with varying degrees of melanosome dispersion; however, we cannot rule out that amino acid variations could cause a slight change in receptor binding (e.g., with the *N*-terminus not included in determined structures or in the half-life of the synthetic peptides).

## 3. Discussion

In this study, we described the development and validation of a biological assay used to test the pharmacological efficacy of α-MSH-derived compounds. In several clinical trial and animal model studies, α-MSH-based peptides have shown potential as a pharmacological therapeutic for the core behavioral symptoms of ASD. The development of an effective pharmaceutical option treating core behavioral symptoms would be a highly valuable, yet currently unavailable, treatment option for caregivers to help manage this challenging disorder. Therefore, an investigative tool like the melanophore assay would contribute toward this end by discovering new, efficacious candidates for ASD therapeutics development.

The investigation into naturally-occurring derivatives of α-MSH found in different fish species demonstrated the sensitivity of the assay method, particularly for compounds registering lower physiological potencies. Although derivatives showed a lower effect than that of the human form, the differing outcomes rather supported the accuracy of the assay protocol. Toward this end, we intentionally selected and designed α-MSH peptide sequences to differ one from the other—e.g., in the number of variant sequences (as with zebrafish and carp) and/or peptide length (cavefish and salmon). Cavefish and salmon sequences comprised opposite ends of the spectrum for dispersive characteristics. Cavefish recorded the lowest dispersive effect out of the four fish species. Interestingly, cavefish lack eyes as well as skin pigmentation, perhaps due to a lack of utility in a cave environment. The melanocortin system, namely α-MSH and MC1R, is the main regulatory mechanism of skin pigmentation in fish and humans [27,28]. Thus, it follows reasonably that the physiological activity of cavefish α-MSH is largely reduced compared with that of other species which make more robust use of skin pigmentation mechanisms. Salmon α-MSH, on the other hand, induced the highest melanophore dispersion of all fish species, bordering on human α-MSH levels. Based on amino acid classification, the branched and nonpolar isoleucine residue most closely matches that of the compact human valine residue, while the long, straight-chained methionine is rather different and more flexible. In any case, our insights from modeling studies at a zebrafish MC1R/α-MSH complex (Figure 6) supposed that the Met13 variant is not directly involved in receptor contacts. However, the differential outcomes of α-MSH variants affirmed our original expectation for the melanophore assay to differentially express sequence variants of α-MSH-based peptides, while the molecular reason of this discrimination in the assay needs further investigation—e.g., regarding the half-life or solubility of various peptides. 

The basic tenet of the melanophore assay was that the pharmacological effect of a compound on a human/animal subject could be ascertained by the degree of melanophore dispersion elicited by the compound. Thus, we sought to validate our protocol with a chemical compound demonstrated to rescue behavioral deficits in ASD individuals/animal models. The literature search yielded a study by Minakova et al. [7] which demonstrated that MT-II, a synthetic α-MSH analogue, rescued social behavior deficits in ASD mice. The results from the melanophore restriction assay demonstrated the superior dispersive power of MT-II compared to human α-MSH. Of note, MT-II differs to α-MSH significantly by a cyclic peptide structure and by a substitution on the core motif of MCR ligand, the HFRW motif (Figure 5a). In MT-II, a D-Phe instead of an L-Phe causes an increased potency of the ligand compared with α-MSH [29]. Together with the cyclic structure compared to the linear of α-MSH, this property should explain why MT-II exhibited the highest potency among derivatives tested here (Figure 4b and Figure 5d). Additionally, the findings reflected the difference in half-life between the two compounds, with the longer-lasting MT-II exhibiting steady, even increasing, melanophore dispersion over time. Considerations regarding half-life also extend to the practical aspect of drug feasibility. Studies have shown that α-MSH, including MT-II, mediates changes in behavior by triggering central oxytocin release [30]; however, oxytocin as a direct therapeutic option encounters several barriers, including BBB impermeability as well as short half-life (3–9 min) [31]. α-MSH and MT-II have well-established BBB permeability; however, MT-II remains more than three times as long in the blood, which would be desirable for treatments of behavioral symptoms. Despite its merits in behavioral research, MT-II is not currently approved to treat any medical condition and may have potentially serious side effects. However, recent U.S. Food and Drug Administration (FDA) approvals of same-family compounds, bremelanotide (2019) and setmelanotide (2020), demonstrate the potential of MT-II and alpha-MSH derivatives as viable pharmacological therapeutics. Thus, the application of MT-II confirmed the sensitivity of the melanophore assay to detect compounds of higher efficacy and provided validation of the assessment of pharmacological efficacy based on melanosome-dispersing capability.

In conclusion, we established a bioassay using the zebrafish animal model by which pharmacological efficacy of α-MSH derivatives could be measured by melanophore dispersion of zebrafish larvae. Clinical as well as animal model studies involving autism spectrum disorder (ASD) have already demonstrated the effectiveness of α-MSH to ameliorate the social burden of autism. Therefore, in order to establish a robust assay method, we identified and analyzed unique sequence variants in naturally-occurring and synthesized α-MSH peptides. In vivo administration of α-MSH derivatives demonstrated both high and low sensitivity of dispersive capability and corresponded to previous findings, e.g., efficacy to improve behavioral deficits in mouse model studies [7]. Our findings showed that MT-II exhibited a higher dispersive capability than human α-MSH, confirming the pharmacological efficacy of highly dispersive compounds. Therefore, we propose the melanophore assay as an investigative tool to screen potential candidates for the development of future α-MSH-based ASD therapeutics.

## 4. Materials and Methods

### 4.1. Zebrafish Husbandry

Larvae used in this study were obtained from matings of wild-type zebrafish from the Zebrafish Center for Disease Modeling (ZCDM; Daejeon, Korea). Fish were raised and maintained under standard conditions [32]. Prior to experiment, all embryos were incubated in egg water, consisting of Sea salts (Sigma Life Sciences, St. Louis, MO, USA) and triple-distilled water, at 28.5 °C until approximately 2dpf.

#### 4.1.1. Melanophore Assay: Dish Protocol

The melanophore assay was initially developed using a single-dish, batch-style protocol which required a mounting step before imaging. Up to five wild-type (WT) larvae were placed in the 13 mm well of a 35 mm confocal dish (SPL Life Sciences, Pocheon-si, Gyeonggi-do, Korea) for administrating reagents. Based on our findings of maximal yolk-sac melanophore development, 48–60 hpf WT zebrafish larvae were randomly selected and added to the confocal well, one dish for each peptide being analyzed or for the control group. Each assay trial was conducted initially with five larval replicates per well; however, in certain cases, some samples either expired or were damaged during the experiment and were excluded from data analysis.

The melanophore dish assay involved three imaging steps and two treatment steps followed by washing (Figure 4a). To begin the experiment, melanophores of the yolk sac region were imaged using either a Leica MZ16 or S6E stereo microscope (Leica Microsystems; Wetzlar, Germany) in order to calculate the initial average melanophore area of the control group larvae. Along with all other experimental groups, the control group was subsequently treated with 50 µL of 1 mM melatonin in 0.1% DMSO (Sigma Life Sciences, St. Louis, MO, USA) to provide the average aggregated melanophore area from which melanosome dispersion by peptide treatment would be calculated. The control group was incorporated into the assay protocol as a reasonable concession between adequate sample size and practical turnaround time—a large portion of which would have been occupied by mounting and imaging procedure for all larvae. We chose to instead determine the initial average dispersed and aggregated melanophore area by a single control group and save this step from each of the other experimental wells, leaving these for final, treatment-induced dispersion images. Therefore, peptide treatment groups began the assay by receiving 50 µL of 1 mM melatonin solution to induce the starting aggregated state of melanophores from which peptide solution was subsequently administered to measure the physiological effect of experimental peptides. Melatonin solution was 50 µL at 1 mM concentration, and peptide solutions were 50 µL at varying concentrations (10, 20, and 40 µM). Peptides were custom synthesized (Peptron, Daejeon, Korea) and their sequences are given in Figure 2b. After melatonin (aggregation) and peptide (dispersion) treatments, the larvae underwent washing (two to three times with triple distilled water) and imaging of yolk-sac melanophores. Details on peptides used in the experiment are provided in the respective subsection below.

#### 4.1.2. Melanophore Assay: Restriction Array Protocol

The second iteration of the protocol saw the introduction of the ZEBRA device and reduction of the mounting steps. The ZEBRA device is a microfluidic restriction array purposed to immobilize zebrafish larvae for quick and convenient imaging over time, while allowing the administration of reagents via access ports directly to the capillary tube (Figure 4b). The restriction assay was used to detail the comparison between two peptide treatments on the dispersive effect over time; 48–60 hpf larvae (*n* = 3–5 larvae) were randomly selected and each was placed in its own capillary tube. Triple-distilled water was used to fill the capillary tube. Melatonin and peptide treatment were administered via access ports at 2 µL volume and varying concentrations (10, 25, 50 µM). Melatonin treatment and peptide treatment times were 10 min and 70 min, respectively. Samples were imaged at the start of the experiment (initial), after melatonin treatment (aggregated), and after peptide treatment (dispersed) at 10-min intervals. Capillary tubes were washed (2–3 times) with triple-distilled water after melatonin and peptide treatments.

### 4.2. Peptides and Reagents

The peptides and reagents used in the melanophore assay are summarized as follows. The melatonin solution used in all assay experiments was prepared by dissolving melatonin (TK) in 0.1% DMSO to a working concentration of 1 mM. The sequences α-MSH peptide from the five different species—*Homo sapiens* “Human” (NP_000930.1 pro-opiomelanocortin preproprotein), *Danio rerio* “Zebrafish” (NP_001076520.1 proopiomelanocortin b precursor), *Cyprinus carpio* “Carp” (KTF79972.1 hypothetical protein cypCar_00032013), *Sinocyclocheilus rhinocerous* “Cavefish” (XP_016411955.1 PREDICTED: pro-opiomelanocortin-1-like), and *Oncorhynchus keta* “Salmon” (XP_035630995.1 pro-opiomelanocortin)—were first obtained via BLAST analysis (https://blast.ncbi.nlm.nih.gov/Blast.cgi; accessed on 27 June 2016) and their custom synthesis was ordered from Peptron (Daejeon, Korea). The purity of synthesized peptides was confirmed by HPLC (>95%). Peptide solutions were prepared by dissolving each peptide in triple-distilled water to a stock concentration and then subsequently diluting to the working concentrations needed for each experiment. Experiments involving MT-II used dilutions of a stock solution consisting of lyophilized MT-II (KoreaPeptide, Seoul, Korea) dissolved in distilled water.

### 4.3. Statistics and Data Analysis

Data collected from the melanophore assay were mainly in the form of photographic images of the larvae yolk-sac region. Images were taken with either a Leica MZ16 or S6E stereo microscope and processed using a combination of computer programs, including Adobe Photoshop (Adobe, San Jose, CA, USA) and ImageJ. Color images were converted into grayscale from which a total of 10 melanophores were randomly selected and individually traced and extracted to calculate the average area of melanophore for the sample at the initial aggregated state (after melatonin treatment) and after dispersion went to completion from α-MSH treatment. GraphPad Prism 5 (GraphPad Software, San Diego, CA, USA) was used for statistical analysis. Protein sequence alignment was performed using Clustal Omega [33] and Jalview [34].

### 4.4. Molecular Modeling of a Receptor-Peptide Ligand Complex

We generated the zebrafish MC1R structure homology model in complex with a zebrafish α-MSH variant based on a human MC4R/NDP-α-MSH complex, determined recently by cryo-EM with 2.9 Å resolution [24]. Several amino acids in the first extracellular loop (EL1) and the intracellular loop three (IL3) are missing in this template structure and were added manually. Amino acids of human MC4R were substituted with corresponding residues of zebrafish MC1R, and NDP-α-MSH residues with zebrafish α-MSH amino acids, respectively. This receptor-ligand complex was minimized by constraining the backbone atoms, followed by molecular dynamics simulation (1 ns) and energy minimization of the side chains (constrained backbone-atoms) until converging at a termination gradient of 0.05 kcal/mol*Å. The entire complex was then energetically minimized without any constraint. The AMBER F99 force field was used for energy minimization and dynamics simulations. For structure preparations, the software Sybyl X2.0 (Certara, Parsippany, NJ, USA) was used and molecular graphics representations were created using PyMol Molecular Graphics System, Version 1.5 (Schrödinger, LLC, New York, NY, USA).

## Data Availability

All data from the present study are available by request from the corresponding author.
